# Regulation of Replication Fork Advance and Stability by Nucleosome Assembly

**DOI:** 10.3390/genes8020049

**Published:** 2017-01-24

**Authors:** Felix Prado, Douglas Maya

**Affiliations:** Department of Genome Biology, Andalusian Molecular Biology and Regenerative Medicine Center (CABIMER), Spanish National Research Council (CSIC), Seville 41092, Spain; douglas.maya@cabimer.es

**Keywords:** DNA replication, chromatin assembly, DNA damage tolerance, replication fork stability, homologous recombination

## Abstract

The advance of replication forks to duplicate chromosomes in dividing cells requires the disassembly of nucleosomes ahead of the fork and the rapid assembly of parental and de novo histones at the newly synthesized strands behind the fork. Replication-coupled chromatin assembly provides a unique opportunity to regulate fork advance and stability. Through post-translational histone modifications and tightly regulated physical and genetic interactions between chromatin assembly factors and replisome components, chromatin assembly: (1) controls the rate of DNA synthesis and adjusts it to histone availability; (2) provides a mechanism to protect the integrity of the advancing fork; and (3) regulates the mechanisms of DNA damage tolerance in response to replication-blocking lesions. Uncoupling DNA synthesis from nucleosome assembly has deleterious effects on genome integrity and cell cycle progression and is linked to genetic diseases, cancer, and aging.

## 1. Introduction

Chromosome duplication during cell division has to accurately maintain the genetic and epigenetic information written in the chromatin. This presents a major challenge for cells, as inferred by the number of mechanisms aimed at preventing and repairing problems generated during DNA replication [1,2]. Defective replication can lead to loss of cell fitness, developmental defects, genetic diseases, and cancer. Indeed, replication-associated genetic instability is linked to the early stages of cancer development [3], and replication stress promotes alterations in the pattern of histone-associated epigenetic marks and might fuel tumorigenesis [4,5]. A major source of genetic and epigenetic instability is generated during the advance of the replication fork, a dynamic nucleosome-free structure with single-stranded DNA (ssDNA) gaps and DNA ends susceptible to being aberrantly processed. These fragile structures have to deal with a number of obstacles such as DNA adducts, abasic sites, DNA-binding proteins and specific DNA and chromatin structures that hamper their advance and compromise their stability, and are therefore specifically controlled by the replication checkpoint to ensure their correct progression and stability.

Chromatin is built by regularly spaced nucleosomes, each of which consists of 146 bp of DNA wrapped around an octamer of two copies each of the core histones H3, H4, H2A, and H2B. The assembly of newly synthesized DNA into nucleosomes occurs during the S phase, with the deposition of an (H3/H4)_2_ tetramer followed by the addition of two H2A/H2B dimers at each side. A key feature of the process of chromatin assembly during DNA replication is that it occurs immediately behind DNA synthesis, with the first deposited nucleosome detected ~250 bp behind the replication fork [6]. The processes of DNA synthesis and nucleosome assembly are physically coupled and genetically co-regulated. In this review, we will focus on how histone dynamics affect replication fork progression and stability, under both unperturbed and stress conditions, and the genetic consequences that the uncoupling between DNA synthesis and histone dynamics at the fork have for genome integrity and cell cycle progression. Therefore, we will introduce only those aspects of replication-coupled nucleosome assembly that are relevant to understanding its connection with replication dynamics. We refer the readers to recent reviews for more detailed analyses of the mechanisms of chromatin assembly, with discussions on the biological functions of canonical and histone variants [7,8,9,10].

## 2. Building a Functional Replisome

**Figure 1:**
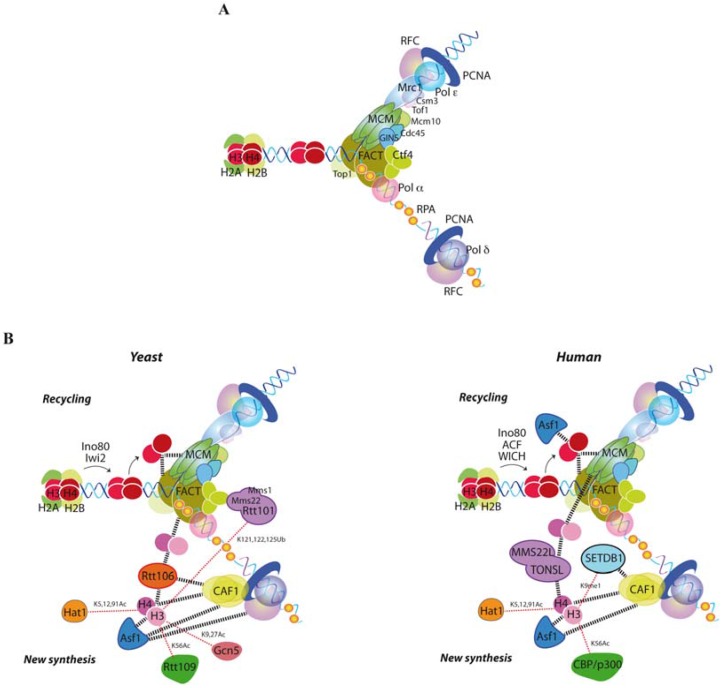
Replication-coupled chromatin assembly. (**A**) Scheme of the eukaryotic replisome progression complex together with the replication processivity factor proliferating cell nuclear antigen (PCNA), its loader replication factor C (RFC), the single-strand (ss)DNA binding protein replication protein A (RPA) and the replicative polymerases. Only the yeast names are indicated here, though all factors are conserved in human [22]; (**B**) Histone modifiers, histone chaperones and histone deposition factors in yeast (left) and human (right). Dashed lines indicate physical interactions. See text for details.

The whole genome has to be replicated once and only once during the cell cycle. This is achieved by temporally separating the loading of the replicative Mcm2-7 helicases onto the origin-recognition complexes (ORC) that mark the replication origins from their further activation. At the end of mitosis, reduction in the levels of cyclin-dependent kinase (CDK) allows the protein Cdc6 to load Mcm2-7 as a head-to-head double hexamer that embraces the double-stranded DNA (dsDNA) molecule. The lack of both CDK and the Dbf4-dependent kinase (DDK) Cdc7 at G1 maintains the helicase inactive until the S phase. During the transition between the G1 and S phase, rising levels of CDK prevent additional Mcm2-7 helicases from being loaded and, together with Cdc7, phosphorylate several subunits of Mcm2-7 and other accessory factors, which then help to recruit Cdc45 and GINS (Sld5/Psf1/Psf2/Psf3). These latter two factors and a single Mcm2-7 hexamer form the CMG (Cdc45/Mcm2-7/GINS) complex, which is the active helicase. At this stage, the two Mcm2-7 helicases are dissociated and encircle opposite ssDNA strands to promote bidirectional replication from each origin [11,12,13] (Figure 1A).

Replication elongation is initiated by DNA unwinding mediated by CMG and requires the DNA synthesis activity of the three multi-subunit DNA polymerases Pol α, Pol δ and Pol ε. A primase-Pol α complex primes continuous DNA synthesis at the leading strand by Pol ε, and discontinuous synthesis of Okazaki fragments at the lagging strand by Pol δ [13,14]. The heterotrimeric ring of proliferating cell nuclear antigen (PCNA), which is loaded at the fork by the clamp loader replication factor C (RFC), is instrumental in this process due to its dual activity as a processivity factor for the polymerases and as a molecular platform that coordinates replication fork advance with chromatin assembly, sister cohesion, and DNA damage tolerance [15].

DNA unwinding and DNA synthesis need to be tightly coordinated to avoid the deleterious effects of an excess of ssDNA at the fork; indeed, ssDNA is coated and protected by the RPA (Replication protein A) complex, and RPA exhaustion causes replication fork collapse [16]. Coupling between the helicase and the polymerases is mediated by Mrc1 and Ctf4, which physically bridge Mcm2-7 with Pol ε, and GINS with Pol α, respectively [17,18], thereby regulating the rate of fork progression [19,20,21]. The replication progression complex comprises the helicase CMG and its activator Mcm10, as well as Ctf4, Mrc1, the replication fork–pausing proteins Tof1 and Csm3, the histone chaperone FACT (Facilitates chromatin transcription) and topoisomerase I [22]. Additional components are also required to replicate the genome, of which some travel continuously with the fork while others are temporarily recruited to overcome specific obstacles. Proteomic analysis of nascent chromatin has recently uncovered the presence of multiple factors involved in DNA repair, checkpoint, cohesion establishment and release of torsional stress [23,24,25].

## 3. Replication Fork Advance through Chromatin

Packaging DNA into nucleosomes could be expected to generate a physical barrier for replication fork advance. Indeed, the activation of the mammalian replication origins is associated with several rounds of abortive DNA synthesis that end up at the boundary of a nucleosome-free region [26], reflecting an early need for chromatin-disrupting activities during replication elongation. Paradoxically, however, the main mechanism required to help forks to progress through chromatin is the nucleosome assembly process itself (Figure 1B).

Chromatin assembly requires a huge amount of histones, which are supplied both by new synthesis and by recycling of parental histones. Subtly reducing the pool of new histones and consequently the degree of nucleosome occupancy accelerates the rate of both RNA synthesis [27] and DNA synthesis [28], and suggests that nucleosomes slow down replication fork advance. This increase in the speed of DNA synthesis, however, could also be associated with the changes in the composition of the parental chromatin that accompany the reduction of canonical histones, such as a drop in the levels of the histone variants H2A.X and H2A.Z and/or an increase in H3.3 [27].

Recycling parental histones not only saves resources but is also an essential mechanism of maintaining epigenetic information. Parental histones are removed from the nucleosomes ahead of the fork and distributed randomly between the sister chromatids [29]. Therefore, the mechanism of histone recycling provides a way to “clear” the road for the advancing replication fork.

The first step for histone recycling is the destabilization of nucleosomes ahead of the fork, which affects an average of two nucleosomes [30]. DNA unwinding by the CMG helicase activity during replication causes an accumulation of positive supercoiling ahead of the fork that might facilitate nucleosome disruption [31]. Whether or not assisted by topological activities, ATP-dependent chromatin remodeling complexes seem to be required for destabilizing the nucleosomes ahead of the fork. In yeast, Ino80 and Isw2 facilitate replication fork progression through a mechanism that becomes less dispensable as the forks move away from the replication origin [32,33,34]. These factors can be detected at stalled forks, although it is not known whether they travel with the fork or are recruited specifically in response to replication stress [32,33,34]. Ino80 is also required for replication fork progression in mammalian cells, in which it interacts with unperturbed forks via the tumor suppressor protein BAP1 and monoubiquitinated histone H2A [35,36]. Moreover, human ISWI facilitates replication elongation as part of the ACF1 and WICH complexes by a mechanism that appears to decondense chromatin [37,38], suggesting that it operates ahead of the fork by breaking higher-order chromatin structures.

A factor that might also connect nucleosome disruption and DNA synthesis at the fork is the histone fold motif-containing protein Dpb4. This protein is an integral component of Pol ε [39] and Iswi2/CHRAC, a yeast counterpart of human ACF [40]. Although its histone chaperone activity has not yet been demonstrated, Iswi2/CHRAC and Pol ε are required for heterochromatin silencing [40].

## 4. Replication Fork Control by Histone Recycling

The mechanisms of histone recycling are still poorly understood. However, it is well documented that the canonical histones H3 and H4 are transferred as a tetramer [29,41,42]. Recent crystal structure analyses have shown that the Mcm2 subunit of Mcm2-7, which contains a conserved region that is able to bind to the globular domain of histone H3 [43,44], can chaperone (H3-H4)_2_ tetramers as part of the helicase alone or with another Mcm2 [45,46]. The physiological relevance of the latter structure is unclear, as only one Mcm2-7 complex is present at the fork. However, we cannot discard that, at some particular regions and/or under specific conditions, the Mcm2-7 helicase at the fork chaperones (H3-H4)_2_ tetramers together with another one from the excess of Mcm2-7 molecules loaded in G1. In any case, these data open the possibility that Mcm2-H3-H4 complexes are intermediates in the process of tetramer transfer during DNA replication [45,46]. In agreement with this possibility, chromatin-bound Mcm2 associates with histone H3 trimethylated at lysine 9 (H3K9me3), a mark of parental chromatin in humans [46], and the histone-binding domain of yeast Mcm2 is required to pick up parental histones that have been released from chromatin [44].

Mcm2 mutations that disrupt its chaperone activity impair cell proliferation in human cells. Indeed, these mutations reduce the binding of Mcm2-7 to Cdc45, establishing a direct connection between histone recycling and replisome activity [46]. In yeast, Mcm2 mutants defective in histone binding are affected in heterochromatin silencing, a phenotype shared by many chromatin assembly mutants. However, they have no detectable defects in bulk replication, in part due to the fact that a second histone chaperone, Spt16, is able to pick up released histones cooperatively with Mcm2 [44]. Spt16 interacts with Pob3 to form FACT in yeast, a conserved complex with roles in transcription, repair, and replication [47]. FACT was identified as part of the yeast replisome progression complex [22], and accordingly, specific physical interactions have been reported between FACT and Pol α and between FACT and RPA in yeast [48,49], and between FACT and Mcm2-7 in yeast and humans [50,51]. In yeast, Spt16 ubiquitination by the ubiquitin ligase Rtt101 positively regulates its interaction with Mcm2-7 [51]. FACT is essential, and genetic analyses in yeast and human viable mutants have shown that it is required for replication elongation [50,52]. Notably, human FACT promotes the unwinding activity of the Mcm2-7 helicase on nucleosomal templates in vitro [50], suggesting that it could directly disassemble nucleosomes ahead of the fork to facilitate DNA synthesis during replication.

Another factor involved in recycling parental histones is Asf1, a conserved histone chaperone first described in yeast for its function in heterochromatin silencing [53] and later purified from Drosophila as a replication-coupled assembly factor (RCAF) [54]. Asf1, which is essential in mammalian cells but not in yeast, has conserved roles in transcription, DNA repair, and replication [55]. Asf1 accumulates at replication foci in *Drosophila* [56] and interacts with RFC in yeast [57] and Mcm2-7 in human cells [58]. Importantly, the histone dimer H3-H4 that bridges Asf1 with Mcm2-7 is specifically modified with parental marks (H4K16Ac and H3K9me3) under hydroxyurea (HU) conditions that enable accumulation of replication forks, suggesting that Mcm2-H3-H4-Asf1 can be an intermediate in the process of parental histone assembly [58]. Interestingly, Asf1 binding to (H3-H4)_2_ splits the tetramer in vitro and binds to the dimer in a way that occludes the H3-H4 tetramerization interface [59,60], and accordingly, a crystal structure of a ternary complex with Asf1, Mcm2 and a dimer of H3-H4 has been solved [45,46]. These results raise the possibility that, under certain conditions, the parental (H3-H4)_2_ tetramer is transiently split during its transfer [61].

Depletion of human Asf1 affects DNA unwinding at replication sites and leads to a reduction in the amount of ssDNA at the fork, and a similar phenotype can be obtained by impairing Asf1 function through histone overexpression [58]. These results suggest that Asf1 might facilitate DNA unwinding at the fork through its capacity to transfer histones during chromatin assembly. Accordingly, Asf1 depletion causes cell cycle arrest in fly, chicken and human cells [56,58,62]. Intriguingly, a V94R Asf1 mutant, which lacks (H3-H4)_2_ splitting activity and cannot form Asf1-H3-H4-Mcm2-7 complexes [58,63], enhances rather than decreases DNA synthesis in a cell-free system of *Xenopus* egg extracts [63]. Asf1 may therefore play a more complex role in the fine-tuning regulation of replication fork progression.

## 5. Mechanisms of New Histone Assembly

In addition to recycled parental histones, chromatin assembly at the fork requires the deposition of newly synthesized histones. Expression of canonical histones is activated in late G1/early S phase to ensure a rapid supply of histones during replication, and it is repressed in early G1, G2, and mitosis to prevent the deleterious consequences of an unscheduled excess of histones for DNA metabolism [64,65]. Accordingly, mutations and inhibitors that impair DNA synthesis trigger a number of mechanisms that repress new histone synthesis and buffer from an excess of histones [66,67].

Newly synthesized histones are chaperoned from the cytoplasm to the nucleus and modified post-translationally to facilitate their transfer to the chromatin assembly factors at the replication fork (Figure 1). In particular, acetylation of the amino terminal tails of H3 and H4 plays redundant roles in chromatin assembly [68]. Acetylation of lysines 5 and 12 in histone H4 by the acetyltransferase Hat1 is conserved from yeast to humans [69,70], while the acetylation pattern of the amino terminal tail of H3 is less conserved. In budding yeast, lysines 9 and 27 are the main targets and are acetylated by the acetyltransferases Rtt109 and Gcn5 [71,72], whereas a fraction of mammalian H3 is acetylated at lysines 14 and 18 [5]. Equally important for replication-coupled chromatin assembly in yeast is the acetylation of H4K91 by Hat1 [73] and of H3K56 by Rtt109 [74,75,76,77]. Indeed, the vast majority of newly synthesized histones H3 is acetylated at lysine 56 in yeast [76]. In contrast, in humans this modification is present in less than 1.5% of histone H3, and marks such as H3.1K9 monomethylation by SETDB1 characterize newly synthesized histones [5].

The contribution of yeast H3K56 acetylation to the deposition of newly synthesized histones has been extensively studied. The chaperone Asf1 plays an instrumental role in this process by binding newly synthesized H3-H4 dimers and presenting them to Rtt109 for acetylation [54,77,78,79,80]. H3K56 acetylation increases the binding affinity of the dimer H3-H4 for the histone deposition factors CAF1 and Rtt106, and the binding of CAF1 to chromatin [78]. This process is facilitated by the Rtt101^Mms1/22^ complex (formed by Rtt101 and the putative adaptor proteins Mms1 and Mms22) or its human ortholog Cul4^DDB1^ [81]. Rtt101^Mms1/22^, which associates with the replication progression complex during S phase [82], binds and ubiquitinates new histone H3 acetylated at lysine 56. This modification weakens Asf1-H3-H4 interactions and facilitates H3-H4 transfer to downstream chromatin assembly factors including Rtt106 [81]. It is important to note that the lack of H3K56 acetylation is not essential, in part due to the Gcn5-mediated acetylation of the H3 amino-terminal tail, which also increases the binding affinity of the dimer H3-H4 for CAF1 and Rtt106 [72].

CAF1 is a highly conserved histone chaperone that operates in the deposition of newly synthesized histones through direct and independent interactions between its three subunits and the H3-H4 dimer [83,84,85,86,87]. Although CAF1 has also been proposed to operate in the recycling of parental histones [9], direct evidence for this hypothesis is still missing. CAF1 is recruited to replication forks through direct interactions with PCNA [88,89,90] and assembles nucleosomes by a mechanism that is stimulated by physical interactions with Asf1 [54,91,92,93]. This CAF1-Asf1 histone deposition complex associates with a single H3-H4 dimer [91], although the capacity of CAF1 to form homodimers might provide the second H3-H4 dimer required for the deposition of a (H3-H4)_2_ tetramer [94]. Moreover, analysis of in vitro interactions has shown that CAF1 can also bind (H3-H4)_2_ tetramers as a monomer [95]. Interestingly, these approaches have shown that H3-H4 binding to Asf1 stimulates the Asf1-CAF1 association, whereas H3-H4 binding to CAF1 is mutually exclusive of Asf1, suggesting a H3-H4 transfer process from Asf1 to DNA via CAF1 [93].

In yeast, CAF1 interacts with the histone chaperone Rtt106 [96]. Rtt106 can also form a homodimer that directly binds to new (H3-H4)_2_ tetramers through a double pleckstrin-homology domain, which interacts with the K56-containing region of histone H3. Indeed, Rtt106-H3 affinity is enhanced upon H3K56 acetylation [97,98]. CAF1 and Rtt106 have redundant roles in the deposition of new histones, and only the absence of both complexes affects the process, although not enough to significantly affect cell growth [78,99]. This points to the existence of additional chromatin assembly activities. Accordingly, FACT is also involved in the deposition of new histones, as supported by the recent purification of a yeast complex formed by FACT, CAF1, Rtt106 and H3K56Ac-H4 (but not Asf1); here, FACT appears to be linked to the complex by Rtt106-H3K56Ac-H4 [99]. The characterization of a histone-interacting defective Spt16 mutant (*spt16-m*) affected in the deposition of new histones has shown that FACT improves both CAF1 and Rtt106 histone deposition pathways; consistently, a triple mutant *cac1∆ rtt106∆ spt16-m* is extremely sick [99]. Newly synthesized histone H2B is also monoubiquitinated at chromatin by the Bre1 ubiquitin ligase at lysine 123, and this modification promotes their assembly or stability [100]. Moreover, H2BK123Ub1 stabilizes Spt16 on replicated DNA, suggesting that this modification cooperates with FACT in histone deposition [100].

In human cells, Mcm2 has been purified with H4K12Ac, suggesting that this chaperone is also involved in the deposition of new histones [46]. Mcm2-7, FACT and Asf1 interact with TONSL, a chaperone without a known homolog in yeast, and its partner MMS22L, the human homologue of Mms22 [101,102,103,104]. Indeed, newly synthesized H3-H4 dimers bridge the interactions between TONSL-MMS22L with Mcm2 and Asf1, suggesting that they form a large histone pre-deposition complex [105].

## 6. Replication Fork Progression and Stability by New Histone Assembly

The aforementioned studies have revealed that a key feature of chromatin assembly is the redundancy of histone deposition pathways. Indeed, impaired histone deposition in CAF1-depleted human cells can partially be compensated by the HIRA-dependent mechanism of replication-independent chromatin assembly, which incorporates the histone variant H3.3 instead of the canonical H3.1 [106]. The presence of these redundant and compensatory mechanisms highlights the importance of ensuring a timely supply of histones during replication-coupled nucleosome assembly.

As shown for the recycling of parental histones, the assembly of newly synthesized histones can strongly influence replication fork advance. In agreement with this, human CAF1 is essential for S-phase progression [107,108]. Likewise, a strong inhibition of histone biosynthesis causes severe defects in nucleosome occupancy and a concomitant reduction in DNA replication in mammalian cells [109,110,111,112,113,114]. This deficit of histones is initially accompanied by an accumulation of PCNA at the forks, suggesting that one way to couple DNA synthesis and histone deposition is by regulating the CAF1-interacting platform [113]. An inverse correlation between the amount of available histones and the length of the S phase has been reported in the fruit fly [115]. These results are consistent with the idea that DNA synthesis is coupled to histone deposition. Indeed, this coupling is actively regulated; for instance, the human protein codanin-1 forms a cytosolic complex with Asf1, H3-H4, and importin-4 in a mutually exclusive interaction with CAF1 and negatively regulates both the amount of Asf1 at chromatin and the rate of DNA synthesis [116]. Along the same line, the response to histone supply is signaled through the Tousled-like kinases, which phosphorylate Asf1 to increase its binding affinity to histones and CAF1 under conditions of limited histone availability, facilitating S-phase progression [117]. In contrast to mammalian cells, partial histone depletion causes only a minor delay in completing S phase in yeast [118]. Likewise, the lack of both H3K56Ac and H2BK123Ub1 only slightly affects completion of DNA replication [100,118,119,120]. An intermediate situation is observed in the fruit fly, which can complete bulk DNA replication in the absence of newly synthesized histones, albeit very slowly [115].

These results suggest that duplication of the more demanding mammalian genome is more sensitive to defects in histone supply. However, accumulation of DNA damage, recombinogenic lesions and gross chromosomal rearrangements (GCRs) in chromatin assembly mutants is observed from yeast to humans [72,74,75,101,103,104,108,113,114,121,122,123], suggesting that the maintenance of replication fork stability is a conserved function of nucleosome assembly. Indeed, replication in *asf1∆* yeast mutants is highly sensitive to the absence of the replicative checkpoints [120]. However, chromatin disassembly/assembly factors are involved in DNA repair, as this process requires access to the lesion and a further restoration of the original chromatin environment, and accordingly, chromatin assembly mutants are sensitive to genotoxic agents [124]. Moreover, many chromatin disassembly/assembly factors operate in other processes that influence genome integrity, such as transcription [55,125]. Therefore, the accumulation of genetic instability in chromatin assembly mutants might stem from defects in the repair of spontaneous DNA lesions and/or DNA processes other than replication.

A connection between nucleosome assembly and replication fork stability is provided by the loss of replisome stability in yeast chromatin assembly mutants under conditions of dNTP depletion by HU as determined by chromatin immunoprecipitation (ChIP) analyses. Thus, Ino80, which accumulates at HU-stalled forks, is required for the stability of RPA, PCNA, and Pol α at the fork [33]. In addition, the lack of either H3K56 acetylation or H2BK123 monoubiquitination causes a drop in the amount of RFC, PCNA, and Pol α the fork [57,80,100]. The lack of H2BK123 monoubiquitination also affects the stability of the CMG helicase and Pol α [100], whereas the lack of H3K56 acetylation does not affect Mcm2-7 binding to the fork but leads to an increase in Pol α and an accumulation of Mcm2-7 ahead of the fork, suggesting that H3K56 acetylation is required to couple DNA unwinding and synthesis [57]. Altogether, these results suggest a role for the assembly of new histones in the stability of stalled replication forks.

**Figure 2:**
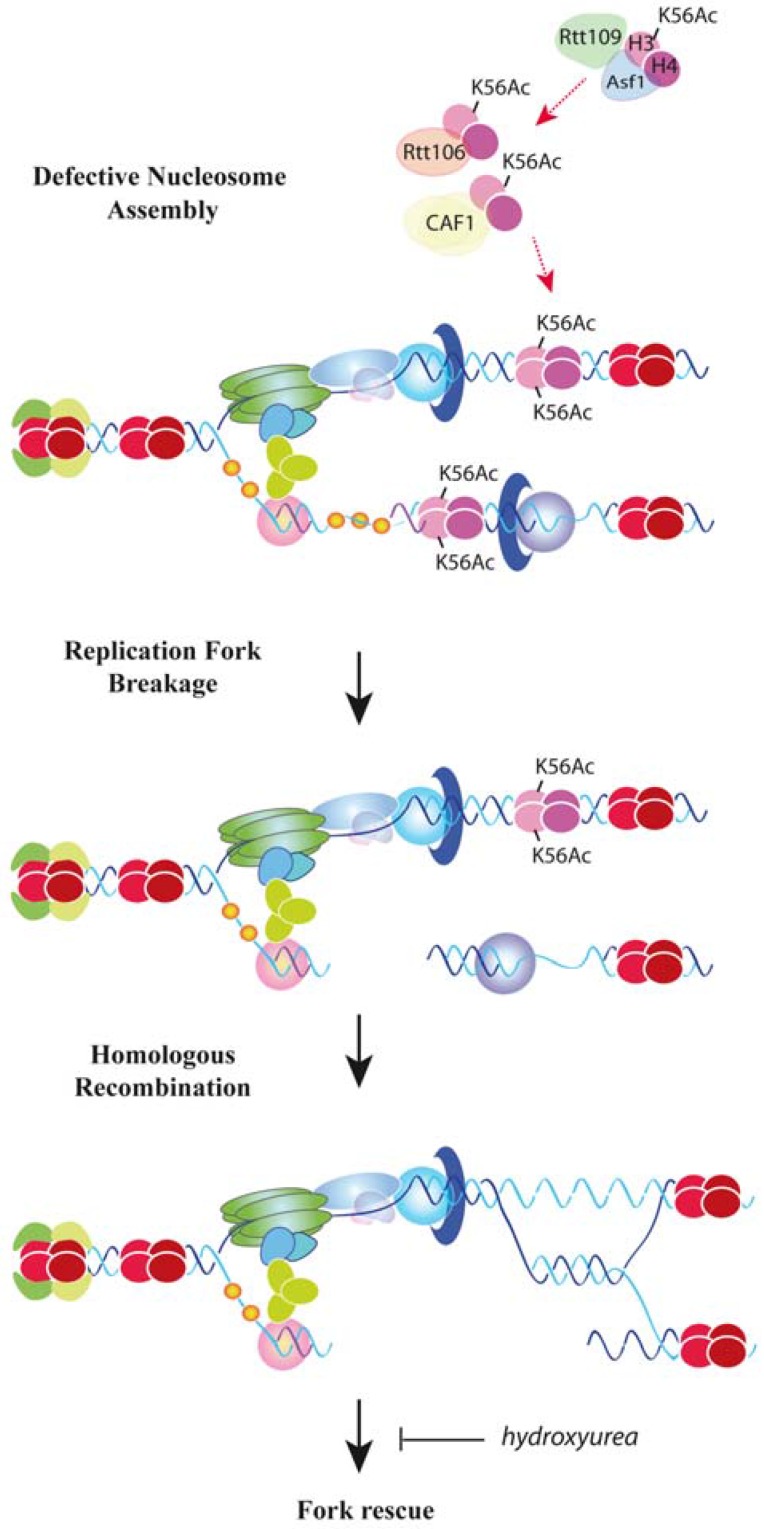
Replication fork stability by chromatin assembly. Defective nucleosome assembly in yeast by a deficit in the pool of newly synthesized histones, or a lack of the H3K56Ac/CAF1/Rtt106 histone deposition pathway (symbolized by a dashed red arrow) causes replication fork breakage and its rescue by homologous recombination [118,119]. Uncoupling DNA synthesis from histone deposition might expose ssDNA fragments to nucleases, impair the process of Okazaki fragment maturation or alter the correct distribution of cohesins, leading to DNA breaks.

How important is chromatin assembly for the stability of advancing forks under unperturbed conditions? ChIP analyses require fork stalling by HU to detect sufficient signal. An alternative approach to avoid this limitation and assess the dynamic and stability of unperturbed advancing forks consists in following the accumulation of replication intermediates from a specific replication origin along a region in synchronized cultures by two-dimensional (2D) gel electrophoresis. Chromatin assembly mutants resulting from partial histone depletion or lack of either H3K56 acetylation (*asf1∆, rtt109∆, H3K56R*) or CAF1 and Rtt106 activities display a strong loss of replication intermediates that is not due to defects in replication initiation or checkpoint-mediated control of fork stability [118,119]. Importantly, the loss of replication intermediates and the subsequent accumulation of recombinogenic DNA lesions and checkpoint activation require the absence of both CAF1 and Rtt106, indicating that these two factors prevent fork collapse by redundant mechanisms [119]. Since *cac1∆ rtt106∆* cells are not impaired in H3K56 acetylation [78], the loss of replication intermediates is due to defective chromatin assembly rather than the absence of H3K56Ac at chromatin. The loss of replication intermediates is similar in *rtt109∆*, *asf1∆*, *cac1∆ rtt106*, and *asf1∆ cac1∆ rtt106∆* mutants, suggesting that the major role of H3K56Ac in fork stability is mediated by its function in nucleosome deposition [119] (Figure 2).

Replication fork instability in yeast cells lacking H3K56 acetylation or CAF1 and Rtt106 activities, or expressing low levels of histones is associated with the formation of recombinogenic structures, and is exacerbated in the absence of the essential recombination protein Rad52. In fact, replication and viability are strongly compromised in these mutants, indicating that defective nucleosome deposition causes fork breaks that are rescued by homologous recombination [118,119]. Accordingly, homologous recombination prevents the accumulation of GCRs induced by the double-strand break (DSB) repair process of non-homologous end joining in H3K56 acetylation mutants [123]. Cells lacking Asf1 are proficient in DSB-induced sister chromatid exchange, a type of event that they accumulate spontaneously [122], suggesting that broken forks in H3K56Ac mutants are repaired with the sister chromatid. Importantly, the loss of replication intermediates in these mutants is higher in the presence than in the absence of HU, except in cells lacking Rad52, suggesting that HU does not destabilize forks in chromatin assembly mutants but rather prevents the rescue of collapsed replication forks by exhausting the pool of dNTP required to resume DNA synthesis [119] (Figure 2). Therefore, nucleosome assembly of newly synthesized histones stabilizes advancing replication forks, and accordingly, yeast chromatin assembly mutants display a genome-wide accumulation of the DNA damage-associated phosphorylation of histone H2A at serine 129 [126].

Nucleosome assembly drives the correct maturation of Okazaki fragments at the lagging strand during replication [127], and yeast mutants defective in Okazaki fragment processing accumulate recombinogenic lesions and GCRs [128,129]. Thus, genetic instability in chromatin assembly mutants might be due to problems in Okazaki fragments’ maturation [130]. The lack of histone H3K56 acetylation in *asf1∆* and *rtt109∆* mutants does not alter the Okazaki fragment periodicity, as observed for mutants lacking CAF1 or Rtt106; however, *asf1∆* and *rtt109∆* share with *cac1∆* and *rtt106∆* cells the accumulation of longer unligated Okazaki fragments, likely due to a delay in histone delivery and nucleosome assembly [131]. This delay could facilitate the accumulation of ssDNA fragments at the lagging strand. In agreement with this, cells lacking Asf1 and Rtt109 accumulate two types of rearrangements that are associated with problems at the lagging strand: CAG/CTG contractions, linked to hairpin-like structures formed at ssDNA regions [132], and ribosomal DNA repeat expansions, likely initiated by fork breakage at the lagging strand [133]. Moreover, *asf1* mutants are highly sensitive to mutations in Pol α and accumulate this polymerase at stalled forks [57,134]. An alternative and not mutually exclusive explanation could be that the loss of replication intermediates in chromatin assembly mutants is due to chromatin condensation, which can lead to breakage of fragile forks in yeast and humans [135,136]. Along the same line, cohesin activity is regulated at the forks by physical interactions between PCNA and yeast Eco1 (ESCO1/2 in humans), an acetyltransferase that modifies cohesins to close the ring and establish sister chromatid cohesion as the fork progresses [137]. Cohesins are required to establish and maintain nucleosome-free regions at intergenic regions [138], and aggravate chromatin defects in histone-depleted cells [139]. Moreover, H3K56 acetylation is required to maintain sister chromatid cohesion, establishing a functional connection between histone deposition and cohesin activity [140]. Further studies are required to understand how chromatin assembly protects replication forks, but it seems clear that DNA synthesis and its assembly into chromatin need to be physically and genetically coupled to maintain genome integrity.

## 7. DNA Damage Tolerance Control by Nucleosome Assembly

Duplicating chromosomes in the presence of DNA lesions that hamper the advance of the replication fork presents a major task for the cell. Cells are endowed with DNA damage tolerance (DDT) mechanisms to bypass the lesion and postpone its repair, thus ensuring timely completion of DNA synthesis. Replication fork lesion bypass occurs by different mechanisms that either directly copy the damaged template using specialized DNA polymerases or circumvent the lesion by switching to the intact sister chromatid template [141,142]. In the latter mechanisms, homologous recombination factors play important roles in facilitating the advance of the replication fork through the DNA lesions by not-yet understood functions [141,143,144,145,146,147,148]. In either case, these mechanisms take place in the context of nucleosome assembly, and it is therefore not surprising to find a growing amount of evidence that supports a direct role of this process in DDT regulation.

H3K56Ac is deacetylated at the end of S/G2 by the sirtuins Hst3 and Hst4, unless cells grow in the presence of DNA damage, which leads to the maintenance of the acetylation by checkpoint-mediated degradation of Hst3/Hst4 [76,140,149,150]. This suggests a role for this modification in the DNA damage response. Indeed, yeast cells defective in H3K56 acetylation are highly sensitive to DNA lesions that trigger the DDT response, namely to methyl-methane sulfonate (MMS)-induced alkylated bases and camptothecin (CPT)-induced Top1-DNA adducts [76]. Genetic analyses suggest that H3K56Ac operates upstream of the Rtt101^Mms1/Mms22^ ubiquitin ligase complex to resist these genotoxic agents [151,152]. As mentioned above, these sensitivities are partially due to the role of chromatin assembly factors in DNA repair processes such as base excision repair and nucleotide excision repair [124]. More direct evidence for a role of H3K56Ac in DDT comes from the specific requirement of Asf1/Rtt109/H3K56Ac and Rtt101^Mms1/Mms22^ to replicate MMS-damaged DNA [152,153,154]. This function is unlikely related to the role of chromatin assembly in fork stability, because cells lacking CAF1 and Rtt106 are much less sensitive to MMS and CPT than H3K56 acetylation mutants, even though they all display a similar loss of replication intermediates [119]. Likewise, mutants defective in the Asf1/Rtt109/H3K56Ac/Rtt101^Mms1/22^ pathway but not in CAF1 Rtt106 are lethal in the absence of the helicase Rrm3 [155], which is required to overcome nonhistone protein-DNA complexes [156]. Along the same line, cells expressing a histone H3K56E mutant are proficient in histone binding to CAF1 and Rtt106 but are sensitive to genotoxic agents [157].

**Figure 3:**
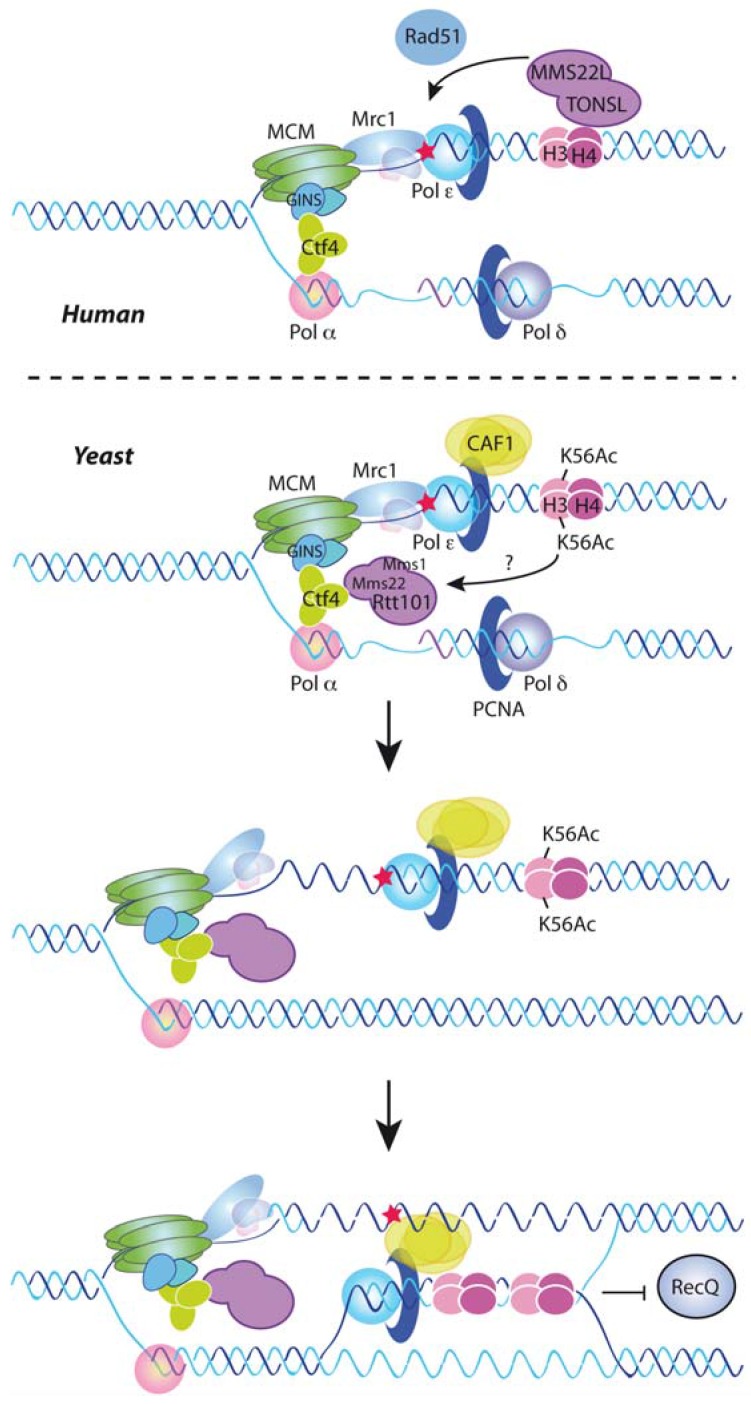
DNA damage tolerance by chromatin assembly. Encounter of a replication fork with DNA lesions that hamper its advance triggers the DNA damage tolerance (DDT) response. In yeast, replication-coupled deposition of newly synthesized histones marks chromatin at the fork with acetylated H3K56, which in turn activates the ubiquitin ligase activity of the Rtt101^Mms1/Mms22^ complex through an unknown mechanism. Ubiquitination of an unknown factor appears to act upon Mrc1 and Ctf4 and uncouple the helicase CMG from the polymerases, facilitating the recombinational bypass of the blocking lesion [82,155]. Moreover, the chromatin assembly factor CAF1 interacts with and counteracts the D-loop dissociation activity of the RecQ helicase, either directly or by assembling nucleosomes onto the D-loop [161]. In human cells, deposition of newly synthesized histones is performed by MMS22L-TONSL, which also binds to unmethylated H3-H4K20 (H3-H4K20me0) and promotes the recruitment of Rad51 and the recombinational rescue of the forks [105].

Components of the Asf1/Rtt109/H3K56Ac/Rtt101^Mms1/22^ pathway are required for the recombinational repair of MMS and CPT-induced replicative DNA lesions but not of DSBs [122,152,153,158]. Consistently, mutants defective in this pathway have problems in checkpoint recovery after drug treatment [119,158]. This suggests a function for this pathway in the template switching mechanism of DDT. Notably, the sensitivity to genotoxic agents and the lethality in the absence of Rrm3 of Asf1/Rtt109/H3K56Ac/Rtt101^Mms1/22^ mutants can be suppressed by mutations in Ctf4, Mrc1, Dpb4, or Mcm6, which uncouple the CMG helicase from the DNA polymerases [82,155]. Moreover, the absence of Mrc1 restores MMS-induced recombination in cells lacking Rtt101^Mms1/22^ [82]. Therefore, H3K56Ac deposition appears to promote the ubiquitination of some unknown substrate to uncouple the replicative helicase from the polymerases as a prerequisite for the recombinational bypass of the blocking lesion (Figure 3, bottom). Consistent with this model, Rtt101^Mms1/22^ physically interacts with Ctf4 though the amino-terminal tail of Mms22, and this interaction is necessary for the function of H3K56Ac in tolerating replicative stress [82,155]. The targets and effects of the ubiquitination are unknown; Mrc1 and Ctf4 are putative targets, but they remain at the forks after DNA damage, indicating that, if targeted, ubiquitination does not lead to their degradation [82,155]. FACT is ubiquitinated by Rtt101 but in an Mms1/Mms22-independent manner [51]. Histones are also potential targets, as they are ubiquitinated in humans by CUL4^DDB^ in response to UV-induced photodimers, and this modification weakens their interaction with DNA and facilitates the recruitment of repair proteins [159]. Finding the target of Rtt101^Mms1/22^/CUL4A^DDB1^ is therefore an essential task for the future for understanding how newly deposited histones facilitate fork progression through DNA lesions.

The human MMS22L-TONSL complex displays remarkable functional similarities with the yeast Rtt101^Mms1/22^ complex. Cells lacking the MMS22L-TONSL complex are highly sensitive to CPT but not to ionizing irradiation-induced DSBs [101,102,103,104]. Moreover, the MMS22L-TONSL complex is required for CPT-induced Rad51 recruitment and homologous recombination, suggesting a role in the recombinational rescue of stressed replication forks [101,104]. Accordingly, the absence of the MMS22L-TONSL complex impairs replication fork progression in the presence of CPT [101,104]. Indeed, MMS22L-TONSL is also necessary for DNA synthesis in the absence of genotoxic agents [104], which likely reflects the requirement of homologous recombination for replication fork progression under unperturbed conditions in mammalian cells [148]. As previously mentioned, less than 1.5% of total histone H3 is acetylated at lysine 56 [5], and this amount does not change during the cell cycle [160]. However, human cells incorporate unmethylated H3-H4K20 histones (H3-H4K20me0) that become methylated in late G2/M [105]. Strikingly, MMS22L-TONSL is able to bind not only to newly synthesized soluble histones, as part of a pre-deposition complex with Mcm2 and Asf1, but also to H4K20me0 at nascent chromatin, where it accumulates in the presence of CPT and facilitates the recombinational response to replicative stress likely by recruiting/stabilizing Rad51 [105] (Figure 3, top).

A direct role for CAF1 in promoting Rad51-mediated replication fork bypass has been recently reported in yeast [161]. Template switching requires Rad51-mediated DNA strand invasion and strand-exchange, which lead to the formation of a D-loop structure that precedes a sister-chromatid junction [141]. D-loop formation and stability are negatively regulated by the dissociation activity of RecQ-type helicases to prevent unscheduled recombination events [162]. Remarkably, CAF1 interacts physically with the *Schizosaccharomyces pombe* RecQ helicase Rqh1 and promotes replication fork bypass by counteracting D-loop dissociation by Rqh1, suggesting that nucleosome assembly makes the D-loop refractory to the antirecombinogenic activity of Rqh1 [161] (Figure 3, bottom). The physical interaction is conserved between CAF1 and the RecQ-helicase Bloom in human cells, where both factors accumulate at centers of DNA replication in a manner that is stimulated by replicative stress and promotes survival [163].

## 8. Replication-Coupled Chromatin Assembly and Disease

Alterations in genome integrity and chromatin structure are something frequently observed in a large number of diseases including neurological and developmental disorders [164] and cancer [165], as well as during senescence and aging [166,167]. In this review, we provide strong evidence for concluding that chromatin assembly defects during replication can cause replication stress and DNA damage. Uncoupling of DNA replication and chromatin assembly in response to replicative stress can also trigger changes in the epigenome that might fuel cancer [4,5]. In agreement with these alterations in genetic and epigenetic information, enzymes involved in histone metabolism and chromatin assembly are frequently mutated in many diseases and tumors [8]. However, these enzymes play key roles in several cellular processes simultaneously, making it difficult to assess which process(es) are affected in these mutants that are directly involved in the development of each disease.

Key factors involved in chromatin assembly during replication, such as Asf1, CAF1, and FACT, are usually overexpressed (rather than absent or mutated) in tumors and are most likely required for tumor proliferation [168,169,170]. This feature strongly argues in favor of the idea that chromatin assembly during replication constitutes an essential process in which cells are unable to tolerate mutations that interfere with it. Thus, genome instability mediated through defects in nucleosome assembly during replication must arise from mutations that are able to produce minor or transient defects in this process. Here we will specifically highlight some diseases that may be directly linked to defects in replication-coupled nucleosome assembly.

Wolf-Hirschhorn syndrome (WHS) is one of the first examples in the literature of a genetic disorder that might be associated with a defect in chromatin replication. This clinically variable and complex genetic disorder is caused by a partial deletion of the distal part of chromosome 4 (4p16.3), which usually leads to a haploinsufficiency of stem-loop binding protein (SLBP) [171]. SLBP is a key factor involved in histone metabolism and is required for the efficient processing, transport, translation and stability of histone mRNAs [172]. Cell lines derived from WHS patients exhibit a delay in S-phase progression, fewer histones associated to DNA, and a higher amount of soluble histones associated to histone chaperones [171]. SLBP depletion recapitulates all these phenotypes observed in cell lines from WHS-patients [113]. This feature suggests that chromatin assembly defects during replication may be directly involved in the development of this syndrome. Interestingly, SLBP has quite recently been shown to be a target of arsenic, a common carcinogenic agent that promotes genome instability [173,174].

Viral infections are also strongly connected to changes in host chromatin and may trigger genome instability through a negative regulation of histone biosynthesis. Many viral pathogens need to change and modify chromatin structure in order to facilitate processes such as transcription, integration, replication, and latency. These viruses usually secrete proteins that either directly change the chromatin structure or that modify the function of host proteins, which will then accomplish this function [175]. Tax is a viral protein secreted by the human T-cell leukemia/lymphotropic virus type-I (HTLV-1) that acts as an oncogene and promotes adult T-cell leukemia/lymphoma (ATLL). Tax expression in cells leads to genome instability and promotes the formation of multinucleated giant cells, in a process that is thought to facilitate ATLL, but that remains poorly understood. Notably, Tax can target and decrease the amount of histones present in the cell during DNA replication and has been proposed to be an enhancer of genome instability upon HTLV-1 infection [176]. HIV infection also enhances genome instability and promotes the formation of several types of lymphomas [177]. Interestingly, a recent report from patients infected with HIV has inversely correlated the ability of this retrovirus to self-replicate with the levels of SLBP present in the cell [178].

Congenital dyserythropoietic anemia type I (CDAI) is another disease recently connected to replication-coupled chromatin assembly. This rare anemic disorder is caused by mutations in the gene that encodes codanin-1, *CDAN1* [179]. Codanin-1 interacts with Asf1 in the cytoplasm through the same region as CAF1 and this interaction presumably regulates the amount of Asf1 at chromatin and the rate of DNA synthesis. Indeed, a conditional cell line expressing a codanin-1 R714 mutant, which is present in some CDAI patients, is unable to maintain Asf1 in the cytoplasm, providing a possible explanation for how mutations in CDAN1 promote this disease [116].

Recent evidence in the literature also links chromatin replication to telomere maintenance in cancer. Alternative lengthening of telomeres (ALT) constitutes an alternative pathway to telomerase reactivation present in approximately 10% of tumors and is thought to preserve telomere length through a recombination-dependent mechanism [180]. Asf1 depletion promotes ALT in a process that exclusively takes places in cells with long telomeres and that requires a functional intra-S-phase checkpoint. Uncoupling of chromatin assembly and DNA replication in the absence of Asf1 appears to impair replication through telomeres leading to fork collapse and hyper-recombination [181]. Asf1 depletion constitutes the first reported case of a direct induction of ALT in human cells, which strongly argues in favor of chromatin replication playing a central role in the generation of ALT. However, Asf1 mutations are rare in cancer suggesting either that ALT formation in tumors is not related to a defect in Asf1 function or that other mutations can target Asf1 function indirectly.

Finally, a connection between chromatin assembly and replicative senescence has been uncovered with the observation that cells, from yeast to human, induce histone depletion during telomere shortening [182,183,184]. In human cells, histone depletion is accompanied by down-regulation of SLBP, CAF1, and Asf1 and leads to a loss of nucleosome occupancy and epigenetic information at telomeres, which activates the DNA damage response and accelerates the program of replicative senescence [183]. Likewise, old yeast and quiescent skeletal muscle stem cells have reduced levels of canonical histones [185,186], which lead to transcriptional misregulation and genome instability [187].

## 9. Concluding Remarks

An integrative view of DNA replication cannot be presented without taking into consideration the process of nucleosome assembly. The main players of DNA synthesis and histone deposition have been biochemically defined. We now describe several examples of the importance of chromatin assembly for replication fork progression and stability through undamaged and damaged templates. Although we have focused on how H3-H4 assembly influences replication fork progression and stability, it is highly likely that the dynamics of both H2A-H2B and histone variants also have a direct impact on the replication process. Indeed, we cannot establish to what extent the replication defects associated with mutations in FACT are related to its function as a chaperone of H2A-H2B dimers [47]. The same can be argued for the reported replicative defects in cells depleted of canonical histones [109,110,111,112,113,114,115,118].

Further advances in this field demand a direct approach to determine the mechanisms by which cells sense histone availability and signal this information to the replication apparatus, to adjust replication fork speed to histone supply. Nucleosome stability maintains the integrity of advancing replication forks, but the functional mechanisms remain elusive. Likewise, we are just starting to appreciate the complexity and relevance of newly assembled chromatin structures during DNA damage tolerance (DDT). In all these cases, global and local factors are likely to play important roles. Indeed, some aspects of the process of replication-dependent chromatin assembly have not been discussed here because there is still little evidence connecting them to the advance and stability of the replication forks. These include the timing of chromatin duplication (early, middle, or late S phase), the asymmetric inheritance of parental histones (and therefore of epigenetic information) at specific loci, the local chromatin states (e.g., euchromatin versus heterochromatin or loci-specific chromatin marks), and the physiological context (e.g., development) [10]. A detailed understanding of these processes may be particularly relevant to revealing the functions of nucleosome assembly in the replication of the more demanding genomes of higher eukaryotes. Finally, it is also worth mentioning that although we have focused on how chromatin assembly regulates DNA replication, the influence is reciprocal. For instance, replication stress generated by replisome impediments facilitates the formation of heterochromatin [188]. The combination of genetic and biochemical approaches with genome-wide analyses may help to unveil the kinetics of chromosome duplication under these different scenarios, and to understand the molecular basis of how defective replication-coupled chromatin assembly contributes to genetic diseases, cancer, and aging.
